# Biological Effects of a *De Novo* Designed Myxoma Virus Peptide Analogue: Evaluation of Cytotoxicity on Tumor Cells

**DOI:** 10.1371/journal.pone.0024809

**Published:** 2011-09-19

**Authors:** Taghrid S. Istivan, Elena Pirogova, Emily Gan, Nahlah M. Almansour, Peter J. Coloe, Irena Cosic

**Affiliations:** 1 School of Applied Sciences, Science Engineering and Health College, RMIT University, Melbourne, Australia; 2 School of Electrical and Computer Engineering, Science Engineering and Health College, RMIT University, Melbourne, Australia; 3 Health Innovations Research Institute, RMIT University, Melbourne, Australia; Leeds Institute of Molecular Medicine, United Kingdom

## Abstract

**Background:**

The Resonant Recognition Model (RRM) is a physico-mathematical model that interprets protein sequence linear information using digital signal processing methods. In this study the RRM concept was employed for structure-function analysis of myxoma virus (MV) proteins and the design of a short bioactive therapeutic peptide with MV-like antitumor/cytotoxic activity.

**Methodology/Principal Findings:**

The analogue RRM-MV was designed by RRM as a linear 18 aa 2.3 kDa peptide. The biological activity of this computationally designed peptide analogue against cancer and normal cell lines was investigated. The cellular cytotoxicity effects were confirmed by confocal immunofluorescence microscopy, by measuring the levels of cytoplasmic lactate dehydrogenase (LDH) and by Prestoblue cell viability assay for up to 72 hours in peptide treated and non-treated cell cultures. Our results revealed that RRM-MV induced a significant dose and time-dependent cytotoxic effect on murine and human cancer cell lines. Yet, when normal murine cell lines were similarly treated with RRM-MV, no cytotoxic effects were observed. Furthermore, the non-bioactive RRM designed peptide RRM-C produced negligible cytotoxic effects on these cancer and normal cell lines when used at similar concentrations. The presence/absence of phosphorylated Akt activity in B16F0 mouse melanoma cells was assessed to indicate the possible apoptosis signalling pathway that could be affected by the peptide treatment. So far, Akt activity did not seem to be significantly affected by RRM-MV as is the case for the original viral protein.

**Conclusions/Significance:**

Our findings indicate the successful application of the RRM concept to design a bioactive peptide analogue (RRM-MV) with cytotoxic effects on tumor cells only. This 2.345 kDa peptide analogue to a 49 kDa viral protein may be suitable to be developed as a potential cancer therapeutic. These results also open a new direction to the rational design of therapeutic agents for future cancer treatment.

## Introduction

In recent years, viral therapy has proved to be successful in cancer treatment, as many viruses were reported to have a positive effect on tumor regression [1], [2]. The ideal oncolytic virus candidate is required to possess a selective tropism for tumor tissue, and little or no ability to cause significant disease in normal tissue [2], [3]. The adenovirus, herpes virus, reovirus, measles virus, poxvirus and myxoma virus were shown to have varying degrees of oncolytic efficacy in numerous tumor models [4], [5], [6], [7].

The myxoma virus (MV) is a rabbit-specific poxvirus pathogen of the *Leporipoxvirus* genus. It causes a lethal disease known as myxomatosis in European rabbits but is not known to cause disease in other vertebrates. Therefore it was used to control the feral rabbit population in Australia in the last century [8]. MV can infect non-rabbit cells *in vitro* including immortalized baby monkey kidney fibroblasts, primary murine cells genetically deficient in interferon responses such as B16 mouse melanoma cell line, and a number of different human tumor cells [9], [10]. It has been considered as an attractive oncolytic agent against human cancers such as human malignant glioma *in vitro* and *in vivo* [6], [11]. A recent study [7] demonstrated that MV is capable of targeting and destroying tumors while causing no significant disease or collateral tissue infection in an immunocompetent host.

The ability of MV to replicate in human cancer cells has been linked to the hyperactivation of an enzyme called serine/threonine kinase Akt in cancer cell lines and to a viral ankyrin-repeat protein NM-T5 (or M-T5, GenBank: AAC55050) which binds to Akt, and promotes its phosphorylation and activation in permissive cancers. NM-T5 is a stable, 49 kDa, cell associated protein encoded by a particular viral host range gene (*M-T5*). This gene is 1452 nucleotide in length and encodes a protein of 483 amino acids, which is expressed rapidly following infection and remains throughout the course of viral infection [9], [10].

The *M-T5* gene is shown to be required for MV replication in rabbit lymphocytes. It has been suggested that its product (M-T5) specifically promotes MV replication in rabbit lymphocytes by preventing the nonspecific shutdown of protein synthesis, stimulating the induction of apoptosis in these cells [10], [11], [12]. The *M-T5* gene product was reported to be critical for virus replication in the majority of human tumor cells, as an *M-T5* mutant MV was non permissive in human cancer cells which are known to support replication of wild-type MV [9], [10], [12], [13].

In recent years, small molecular weight peptides have been applied into developing cancer therapeutics, mostly for their ability to easily penetrate cellular membranes and to interfere with enzymatic functions or protein-protein interactions within cells [14]. The development of such therapies is focused on small peptides with strong tumoricidal activity and low toxicity aiming at a high therapeutic index on cancer cells and minimizing the undesirable side effects on normal cells [15]. However, the systematic mutation studies aiming to derive biologically active peptides would result in the generation of an enormous number of peptides that must be subsequently synthesized and experimentally tested.

It is generally recognised that the relationship between a protein's structure, its biological function and its abilities to bind to a specific ligand, can be enunciated in terms of a multistage process which involves specific bio-recognition, chemical binding and energy transfer. Property-pattern algorithmic procedures are based on the representation of the primary structure of a protein as a numerical series by assigning a numerical value of a physicochemical parameter to each amino acid. The Resonant Recognition Model (RRM) interprets the linear information of a protein sequence using digital signal analysis [16], [17], [18]. The RRM concept is based on the finding that there is a significant correlation between spectra of the numerical representation of the amino acids and their biological activity. It is assumed that proteins with the same biological function or interactive activity have the same periodic components in the distribution of delocalized electron energies along the protein molecule. It was found that the RRM frequencies represent the characteristic features of proteins' biological functions or interactions [16], [17]. It is proposed that these characteristic frequencies (RRM frequencies) are relevant parameters for mutual recognition between bio-molecules, and are significant in describing the selectivity of interaction between proteins and their substrates or targets but are not chemical binding [19], [20], [21], [22]. The RRM concept was used to predict the hot spot amino acid distribution in primary sequences of the neuropeptide Y family. The study concluded that for the prediction of hot spots, the set of amino acid residues in the N- and C- terminal halves must be conserved equally [23]. The Wavelet Transform (WT) was introduced into the RRM [24] to enable researchers to predict locations of protein active/binding sites directly from analysis of a protein primary sequence. The incorporation of the WT into the RRM was successful for the selected protein examples [22], [24], [25].

In previous studies the RRM approach was applied to structure-function analysis of basic fibroblast growth factor (bFGF) and for analysis of HIV envelope proteins [21], [26]. Property-pattern characteristics for biological activity and receptor recognition for a group of FGF-related proteins were defined and then used to aid the design of a set of peptides which can act as bFGF antagonists. Molecular modelling techniques were then employed to identify the peptide within this set with the greatest conformational similarity to the putative receptor domain of bFGF [21].

The interaction between HIV virus envelope proteins and CD4 cell surface antigen has a central role in the process of virus entry into the host cell. Thus, blocking the interaction between the envelope glycoproteins and CD4 surface antigen, known to be the HIV receptor, should inhibit infection. For this purpose, six peptides, each of 20 amino acids in length, were designed using the RRM methodology. The activities of the designed peptides were evaluated experimentally to validate the RRM computational predictions. The results obtained showed significant cross-reactivity to the polyclonal antibodies raised against peptides that share at least one characteristic frequency and phase at this frequency [26].

In this study we applied the RRM approach [16], [17] to the structure-function analysis of selected MV proteins, and to design a single short linear bioactive peptide that mimics MV-T5 protein activity. The *de novo* designed peptides RRM-MV was assessed for its biological effects in mammalian tumor and normal cell lines. Our results indicated that the RRM concept was successfully applied in the design of a bioactive peptide with a targeted antitumor effect.

## Materials and Methods

### The application of RRM in the design of a short peptide analogue

The RRM is based on the representation of a protein's primary structure as a numerical series by assigning a physical parameter value to each amino acid relevant to the protein's biological activity. The parameter employed in these studies, i.e. electron-ion interaction potential (EIIP) [27], describes a physicochemical property of amino acids (the energy states of valence electrons which are important for interaction between molecules) within a protein sequence. By assigning the EIIP values to the corresponding amino acid in the protein sequence the original protein is converted into the numerical sequence. The obtained numerical series can then be analysed by discrete Fourier transformation, and converted into a discrete spectrum, which carries the same information content about the arrangement of the amino acids in the sequence as the original numerical sequence [16], [17], [18], [28]. Comparative analyses of several hundred proteins and their biological function have shown that: (i) each functional group of proteins exhibits at least one characteristic frequency in their Fourier spectra; and (ii) proteins and their receptors have the same characteristic frequency with opposite phases at this frequency [17], [22], [29]. Hence, from corresponding Fourier Transformation for a particular protein, the amplitude and phase for the specific characteristic frequency can be calculated.

Determination of the characteristic frequency and phase for a selected protein sequence is essential for *de novo* design of bioactive peptide analogues which can mimic the biological activity of a selected parent protein sequence [17], [18], [22], [29]. For this design it is possible to determine the RRM characteristic frequency from the analysis of proteins' power spectra. In addition, the corresponding phase for the particular frequency can be identified from the analysis of their phase spectra. Amino acid sequences (short peptides) having specific characteristics related to the proteins' biological function can be designed on the basis of the determined RRM characteristic frequencies and phases for a particular group of protein sequences. Hence, the designed RRM peptide is expected to exhibit the desired biological activity [17], [21], [22], [26], [29].

### Bioactive Peptide Design Procedure

In this study the RRM approach was utilised for structure-function analysis of myxoma virus (MV) proteins and to design a short therapeutic peptide with MV-like antitumor/cytotoxic activity. The bioactive peptide analogue RRM-MV and the negative control peptide RRM-C were designed using the following strategy for the defined peptide design:

- The RRM characteristic frequency can be determined from the multiple cross-spectral function for a group of protein sequences that share common biological function (interaction).
- The phases for the characteristic frequencies of the protein, which is selected as the parent protein for agonist/antagonist peptide design, were calculated.
- The minimal length of the designed peptide is defined by the appropriate frequency resolution. An Inverse Fourier Transform was used to calculate a numerical sequence of different lengths which exhibits the same prominent characteristic frequency as a parent protein.
- The tabulated EIIP parameter values were used to determine the amino acids that correspond to each element of the new numerical sequence defined above. Thus, the resulting new amino acid sequence presents the anticipated designed peptide [17], [21], [22], [26], [29].

In aiming to design the short bioactive peptide analogue (RRM-MV) ten MV proteins with the following NCBI GenBank codes were analysed using the RRM: M-T1 (NP_051880); NM-T2 (NP_051879); T2 (AAA46632); T3C (CAA09973); MT3 (CAA0997; M-T4 (NP_051716); NM-T5 (AAC55050); M-T6 (CAA09975), NM-T7 (AAA46631); and M-T8 (AAA46630). The 483 aa ankyrin-repeat protein NM-T5 protein (AAC55050) was selected as a parent protein for this analysis.

### Peptide synthesis

The *de novo* designed peptides (RRM-MV and RRM-C) were commercially synthesized to >95% purity by AUSPEP (Melbourne, Australia). Aliquots of the lyophilised peptides were kept at −20°C at all times. Stocks were freshly prepared in Dulbecco Modified Eagle medium (DMEM) and used at the required concentration within one week of preparation.

### Cell lines and culture conditions

Mammalian cancer and normal cell lines were used in this study: mouse skin melanoma B16F0 (donated by Dr. Glen Boyle, QIMR, Australia), mouse macrophage J774 cell line and Chinese hamster ovary CHO cell line (our laboratory's stock cultures), the wild type mouse skin fibroblasts primary cell culture, and human squamous cell carcinoma (COLO 16) cell lines (donated by the School of Medical Sciences, RMIT University). All cell lines are adherent except J774 which is a semi adherent cell line.

Cell cultures were grown in complete DMEM (invitrogen, Oceania) except COLO 16 which was maintained in Roswell Park Memorial Institute medium (RPMI) 1640 (invitrogen, Oceania). All cell cultures were supplemented with 10% heat-inactivated foetal bovine serum (FBS) (Bovogen Biologicals, Australia) and kept at 37°C in a humidified 10% CO_2_ incubator. No antibiotics were used in growing and maintaining the cell cultures. Cell lines were regularly screened to ensure they remained Mycoplasma-free using Mycofluor Detection Kit (invitrogen, Oceania).

### Treatment of cell cultures with RRM peptides

Cells were seeded (3×10^5^ cells per well) and grown to 90–95% confluency in 24 well plates before treatment with the bioactive peptide analogue RRM-MV or with the negative control peptide RRM-C. Cell cultures, not exposed to any of the peptides, were also included in all assays as no-treatment controls (blank). Each peptide was freshly dissolved in DMEM and then added to the 95% confluent cell culture in final concentrations of 50 ng/ml (21.32 nM RRM-MV or 20.37 nM RRM-C); 100 ng/ml ( = 42.6 nM RRM-MV or 40.7 nM RRM-C); 200 ng/ml ( = 85.3 nM RRM-MV or 81.5 nM RRM-C); 400 ng/ml ( = 170.5 nM RRM-MV or 162.9 nM RRM-C); 800 ng/ml ( = 341 nM RRM-MV or 325.9 nM RRM-C) and 1600 ng/ml ( = 682 nM RRM-MV or 651.7 nM RRM-C). The cell cultures were further incubated and then checked for cellular changes at intervals of 3 h; 6 h, 12 h, and 18 h. Morphological changes in the confluent layer of each cell culture were initially compared with both the negative and no-treatment controls using microscopic examination. All samples were tested in duplicates and each test was repeated at least three times.

### Detection of apoptosis and necrosis by confocal immunofluorescence microscopy

Cellular apoptosis and necrosis were investigated using Vybrant Apoptosis Assay kit #2 (invitrogen, USA) which contains Annexin V-Alexa Fluor 488 (AF488) conjugate and Propidium Iodide (PI). Cell cultures in growth medium (without FBS) were treated with selected concentrations of the peptides and re-incubated for (3 h–16 h) to detect the dose-dependent effect of the peptides. After incubation, cell cultures were washed once with ice-cold 1× PBS and labelled with annexin V-AF 488 and PI according to the manufacturer's instructions with slight modifications. Briefly, cells were incubated at room temperature for 20 min with annexin V binding buffer (10 mM HEPES; 140 mM NaCl; 2.5 mM CaCl_2_, pH 7.4) containing 5 and 1.5 µL of the conjugate and PI, respectively. They were then washed twice and resuspended in a binding buffer for further analysis. Confocal laser scanning microscopy (CLSM) was carried out with a Nikon Eclipse Ti-E A1 laser-scanning confocal system (Nikon Instruments Inc, USA), using 10×, 20× and 40× objectives. In order to compare the extent of apoptosis between treatments, the pinhole aperture and other settings were fixed. Cell images captured were analysed with the NIS-Element imaging software.

### Quantitative assessment of the peptide treatment

#### Detection of cellular cytotoxicity by LDH assay

Cell cytotoxicity was assessed by measuring the release of cytoplasmic lactate dehydrogenase (LDH) into cell culture supernatants. Cell cultures were seeded and grown as previously indicated and then incubated with specific concentrations of the peptides at 37°C for 3 h. LDH activity was assayed using the Cytotoxicity Detection Kit (Roche Diagnostics, USA) according to the manufacturer's instructions. Experiments were performed in triplicates with three repeats for each experiment. The percentage of cell cytotoxicity was calculated using the following formula: 100×[(experimental LDH release-spontaneous LDH release)/(maximum LDH release-spontaneous LDH release)], as shown in the manufacturer's protocol.

#### Detection of cellular viability after peptide treatment

Cell survival in samples after treatment was measured using Prestoblue™ Cell Viability reagent (invitrogen, USA) according to the manufacturer's protocol. Data values were measured as OD readings at 570 nm after addition and incubation with the reagent. All samples were tested in triplicates and each test was repeated at least three times. Cell viability was calculated using the following formula: 100×[(OD_570_ of treated sample)/(OD_570_ of untreated sample)].

#### Single dose effect

Cells seeded at a density of 1×10^5^ cells per well in 96 well plates were treated with the bioactive peptide analogue RRM-MV or with the negative control peptide RRM-C. Cell cultures (untreated with any of the peptides and treated with 60% DMSO) were also included in all assays as no-treatment (blank) and positive controls, respectively. Each cell line was incubated *in vitro* for 8 h; 16 h; 24 h; 48 h and 72 h with DMEM media containing (50 ng/ml; 100 ng/ml; 200 ng/ml; 400 ng/ml and 800 ng/ml) of RRM-MV and 400 ng/ml of RRM-C. Cell viability was assessed as indicated above.

#### Repeated dose effect

Cells seeded at a density of 1×10^5^ cells per well in 96 well plates were treated as indicated above for 16 h. A second dose of DMEM with RRM-C or RRM-MV or DMSO (positive control) was added after the 16 h treatment and cell cultures were then incubated further for another 10 h; 24 h and 48 h. Cell viability was assessed as indicated above.

### Detection of p-Akt and total AKT by Immunoblotting

Western blots (WB) to detect total Akt and phospho-Akt (p-Akt) were carried out on B16F0 cells as described in [9], with minor modifications. Cells were seeded at a density of 7×10^5^ per well and were serum-starved overnight, then they were either treated with 50 µM PI3 kinase inhibitor LY294002 (Cell Signalling Technology, USA) for 1 h, or were treated with 800 ng/ml of RRM-MV or RRM-C for 3 h. Whole cell proteins were then extracted as described above, separated by SDS-PAGE and transferred onto nitocellulose membranes. WBs were probed separately either with rabbit pan-Akt monoclonal antibody (MAB) (Cell Signalling Technology, USA) to detect total Akt or with rabbit phospho Akt (Thr308) MAB (Cell Signalling Technology, USA), to detect phosphorylated Akt at a 1∶1000 dilution. An alkaline phosphatase-conjugated goat anti-rabbit polyclonal antibody (Sapphire Bioscience, Australia) was used as a secondary antibody at a 1∶5000 dilution and detected colorimetrically with BCIP/NBT substrate (Amresco, USA). WBs were repeated three times with cell cultures and cell lysates prepared on different days.

### Statistical analysis

Statistical analysis on cellular cytotoxicity data and on cell viability data was conducted with one-way ANOVA and Dunnett's test, which compares the means of all treatments with a designated control (e.g. negative control peptide or untreated cells).

## Results

### Computational analysis of MV proteins and *de novo* peptide design

**Figure 1:**
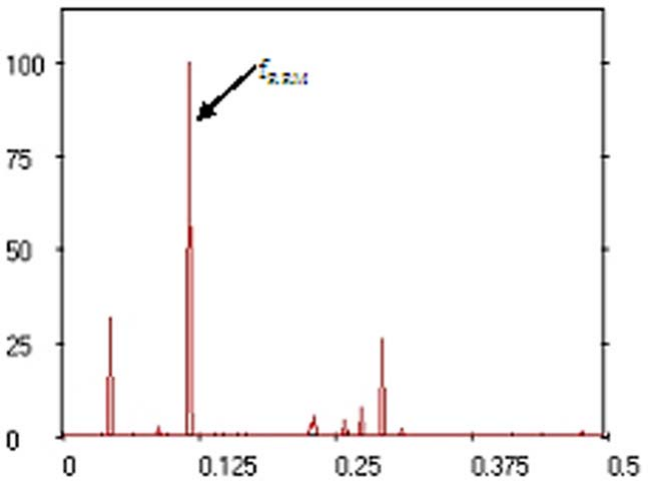
Multiple cross-spectral function of myxoma virus proteins (10 sequences alignment).

The RRM characteristic frequency of the selected MV proteins was identified at f_RRM_ = 0.1152 (Figure 1). According to the RRM concepts, this prominent peak characterises the common biological activity of the analysed MV proteins. Less prominent peaks observed in Figure 1 indicate that these selected MV proteins can be involved in different biological processes (i.e. interact with other proteins). As a result, the 18 aa linear peptide sequence (MDDRWPLEYTDDTYEIPW) for RRM-MV was designed with the frequency f_MV_ = 0.1152 and phase ϕ_MV_ = −0.457. ProtParam (http://au.expasy.org/tools/protparam.html) was used as a tool for the computation of physical and chemical parameters for the RRM designed peptide sequences. RRM-MV predicted MW is 2.345 kDa; theoretical pI: 3.66; estimated half-life in mammalian reticulocytes: 30 h; and instability index: 27.32 which classifies the protein as stable.

The RRM procedure was also used to design the negative control peptide (RRM-C), which has different inactive frequency and phase (f_C_ = 0.2 and phase ϕ_C_ = 1.5) and as assumed, it would not express MV-like cytotoxic activity. The 22 aa linear peptide (CVLQDCVLQDCVIQDCVLQDCV), was designed as a negative control for the biological cytotoxicity assays. RRM-C predicted MW is 2.454 kDa, theoretical pI: 3.32, and estimated half-life in mammalian cells is 1.2 h (ExPASy - ProtParam tool).

### Evaluation of cytotoxic effects of RRM designed peptides by confocal immunofluorescence microscopy

#### Effects on cancer cell lines

**Figure 2:**
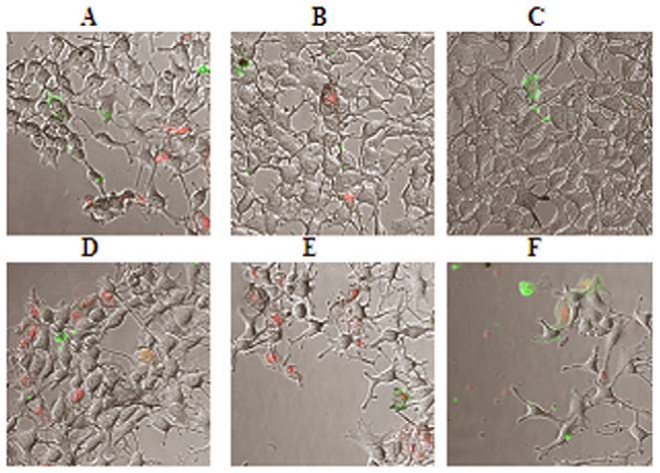
CLSM micrographs for apoptosis/necrosis assay with annexin V-Alexa Fluor 488 (green fluorescence) and propidium iodide (red fluorescence) in mouse melanoma cell line (B16F0). After 3 h incubation with DMEM only in **A** (blank), 3 h incubation with 800 ng/ml RRM-C in **B**, and with 800 ng/ml RRM-MV in C. Cytotoxic changes including detachment of confluent layer, apoptotic cells (green) and necrotic cells (red) and are obvious in **C** when compared with A and B. Longer treatment periods 6 h; 9 h; and 18 h in (**D–F** respectively) with increased levels of necrosis and cellular detachment when B16F0 cell cultures were treated with (800 ng/ml) of RRM-MV. (200× magnification).

The CLSM microscopy results for *in vitro* cytotoxicity assays revealed that the bioactive peptide (RRM-MV) caused noticeable cytotoxic effects (apoptosis and necrosis) on the mouse melanoma B16F0 cell line when compared with B16F0 cell culture treated with the negative control RRM-C and with the non treated B16F0 cell culture (Figure 2 A–C respectively). There was a negligible cytotoxic effect for RRM-C on the B16 F0 cell line (Figure 2.B) when compared with the effect of RRM-MV (Figure 2 A). Longer incubation periods (6 h, 9 h, and 18 h) with RRM-MV induced stronger cytotoxic effects (apoptosis, necrosis and detachment) than shorter incubation (3 h), when the B16F0 cell line was treated with a similar concentration (800 ng/ml) of RRM-MVT5 (Figure 2 D–F). The micrographs indicated detachment of the confluent layer when the cell culture was incubated with 800 ng/ml of RRM-MV for 18 h. In addition, when the B16F0 cell culture was incubated with double dilutions (50 ng/ml to 1600 ng/ml) of RRM-MV for a fixed incubation time (3 h), cellular detachment and cytotoxicity were more significant in wells treated with higher concentrations of RRM-MV (AF488 positive, green apoptotic cells and PI positive red necrotic cells). In contrast, no cytotoxic effect was observed when the B16F0 cell line was similarly treated and incubated with 1600 ng/ml of RRM-C, as compared to the strong cytotoxic effect of 1600 ng/ml of RRM-MV on the same cell line (data not shown). The experimental data presented in (Figure 2) indicate dose- and time-dependent effects of the bioactive peptide RRM-MV, and the absence of any cytotoxic effect after treatment with the negative control peptide RRM-C on the B16F0 mouse melanoma cell line.

**Figure 3:**
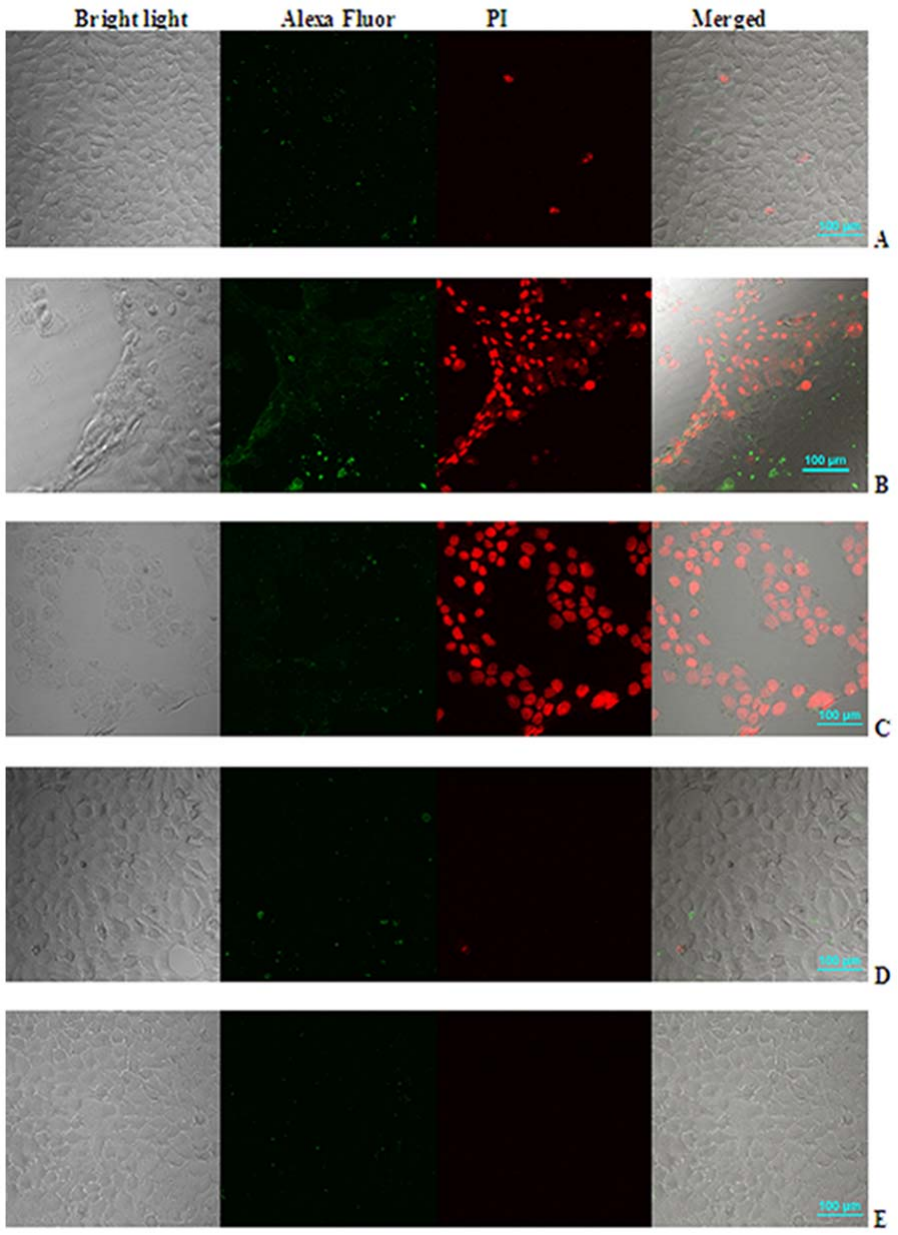
CLSM micrographs for human squamous carcinoma COLO 16 cell line. Cell cultures were treated with 50 ng/ml, 100 ng/ml and 200 ng/ml of RRM-MV for 3 h in **A**, **B**, and **C** respectively. Cell culture in **D** was treated with 200 ng/ml of RRM-C, while cell cultures in **E** were similarly incubated without any treatment. More necrotic cells and detachment can be seen in B and C as compared with A indicating dose-dependent cytotoxic effect of RRM-MV. No cellular detachment can be seen in the cell culture treated with 200 ng/ml of the negative control RRM-C in D or in the non-treated cell culture in E.

The cytotoxic effects of RRM-MV and RRM-C were also evaluated on the human squamous cell carcinoma (COLO 16) cell line. The CLSM data revealed that this human cancer cell line was more susceptible to RRM-MV treatment than the mouse melanoma cell line B16F0. Cytotoxic effects were initially detected when this cell line was treated with 50 ng/ml of RRM-MV for 3 h (Figure 3 A), with few apoptotic cells (green) and necrotic cells (red) being observed. Higher concentrations of RRM-MV (above 50 ng/ml) induced significant apoptosis and necrosis leading to complete detachment of the confluent layer when the cell culture was incubated with 100 ng/ml and 200 ng/ml of RRM-MV for 3 h (Figure 3, B & C respectively), as compared with the effect of 200 ng/ml of the negative control peptide RRM-C and with the non-treated cell culture incubated with DMEM only (Figure 3 D & E respectively). The micrographs in (Figure 3) clearly indicate the dose-dependent cytotoxic effect of the RRM-MV on the human squamous cell carcinoma cell line, and the lack of cytotoxic effect of RRM-C on the same cell line when compared with the non treated cell culture.

#### Effects on normal cell lines

**Figure 4:**
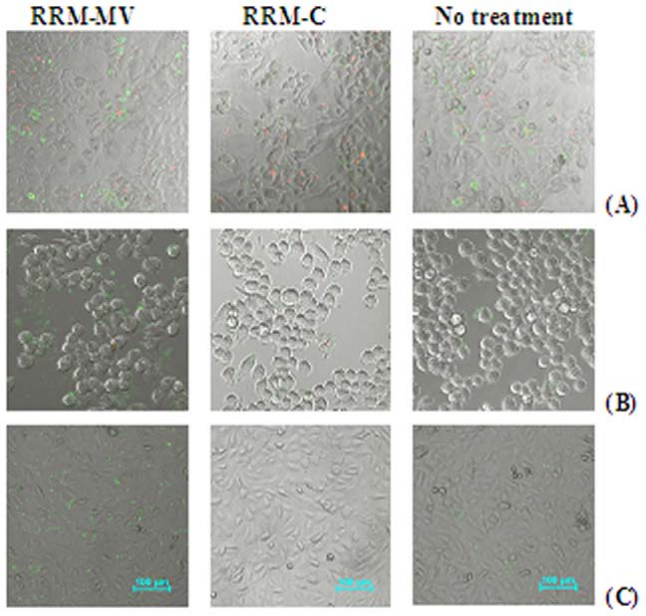
CLSM micrographs for the apoptosis/necrosis assay in three normal cell lines after 3 h incubation with 800 ng/ml of RRM-MV; or 800 ng/ml of RRM-C. Mouse skin fibroblasts in **A**; mouse macrophages J774 in **B**; and CHO in **C**. No significant cytotoxic effects (apoptosis, necrosis and cellular detachment) were detected in all cell cultures treated with RRM-MV or RRM-C as compared with the non-treated cell cultures similarly incubated in DMEM, indicating the minimal cytotoxic effect of RRM-MV on the 3 normal cell lines.

A primary cell culture of mouse skin fibroblast (passage 2) was treated with RRM-MV to evaluate the effect of this bioactive peptide on normal mouse cell lines. The primary culture was incubated for 3 h with two-fold peptide concentrations starting at 100 ng/ml up to 1600 ng/ml of RRM-MV or RRM-C. The cytotoxic effects were assessed by CLSM with the apoptosis/necrosis assay. No significant increases were found in the numbers of apoptotic and necrotic cells in the three normal cell cultures treated with the bioactive peptide RRM-MV, even at the highest cytotoxic dose of 1600 ng/ml, when compared with the negative control peptide RRM-C and with non-treated cell cultures (Figure 4 A, B & C). It is obvious from the CLSM micrographs in this figure that RRM-MV did not induce any significant cytotoxic changes on the normal skin cell culture from the number of apoptotic cells (green) and/or necrotic cells (red) in the treated and non-treated normal skin cell cultures (Figure 4 A), as compared with the cytotoxic effects of RRM-MV on the two cancer cell lines (Figures 2 &3).

The effects of RRM-MV and RRM-C on the semi-adherent mouse macrophage cell line J774 were similarly investigated to evaluate the cytotoxicity of these peptides on murine macrophages. The microscopic images for J774 shown in (Figure 4, B), indicate the absence of cytotoxic effects of the RRM-designed peptides on the macrophages, although an increase in the green fluorescent intensity in cell cultures treated with higher concentrations (800 ng/ml and 1600 ng/ml) of RRM-MV was observed. Necrotic macrophages were not detected in J774 cultures treated with RRM-MV, indicating the absence of cytotoxic effects on these normal mouse cells as compared to significant cytotoxic effects induced by similar concentrations of this peptide on B16F0 mouse melanoma cell line (Figures 2 and 4, B).

To confirm the negligible cytotoxic effect of RRM-MV on normal cells, another normal transformed cell line CHO was treated with RRM-MV or with RRM-C. The cytotoxic effects of the RRM-designed peptides on the CHO cell line were similar to the effects on the mouse macrophage cell line, as necrotic cells were not detected in cell cultures treated with either RRM-MV or RRM-C as shown in the CLSM micrographs (Figure 4, C).

### Quantitative assessment of peptide treatment

#### Detection of cellular cytotoxicity on cancer and normal cells by LDH assay

**Figure 5:**
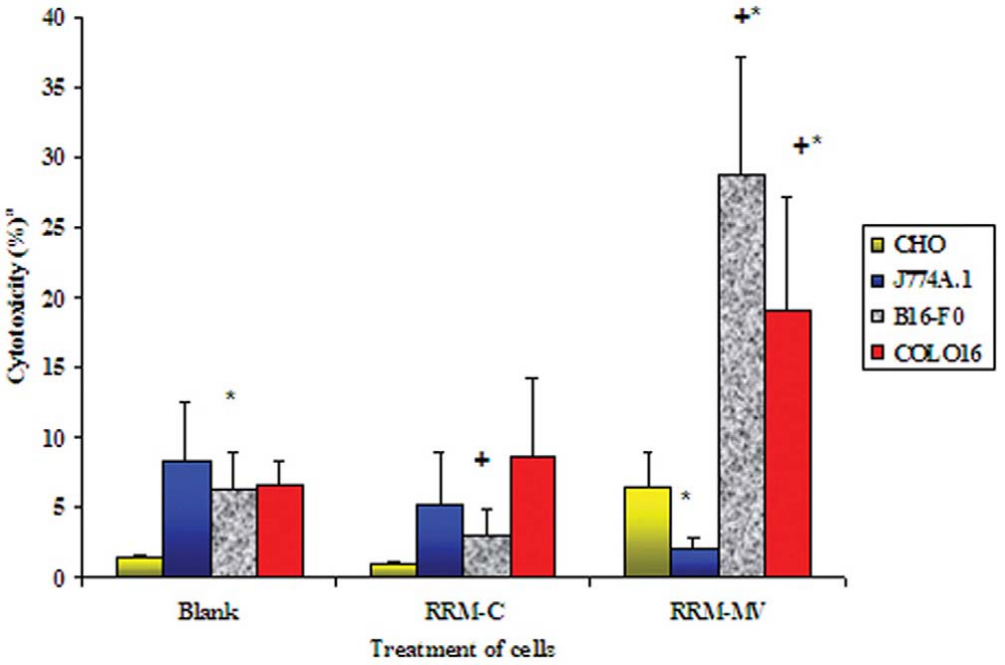
Cytotoxic effect of RRM peptide analogues on normal and cancer cells measured by LDH assay. Cells (3×10^5^) were incubated for 3 h with control peptide (RRM-C), or with RRM-MV at 400 ng/ml (for COLO16) and 1600 ng/ml (for CHO, J774A.1 and B16-F0). Cells without treatment were similarly incubated for 3 h (blank). Each bar represents mean ± standard errors of 3 separate experiments in triplicate. Data values that are significantly altered (ANOVA and Dunnett's post-hoc analysis) are indicated by * (when compared to control treated cells) and **+** (when compared to untreated cells) at a significant level of *p*<0.05.

When tested by the LDH quantitative assay, RRM-MV concentrations of 1600 ng/ml and 400 ng/ml had a significant cytotoxic effect on the mammalian cancer cell lines B16F0 and COLO16 respectively, leading to high LDH release and cytotoxicity (Figure 5). Treatment of B16F0 cells with RRM-MV produced significantly higher LDH levels when compared to both untreated (blank) and RRM-C-treated cells. Conversely, RRM-MV had no cytotoxic effect on both the mouse macrophage J744 and the CHO cell lines when compared with the non-treated cultures (Figure 5). The LDH cytotoxicity experimental data for the peptide treatment of cancer and normal cell lines in figure 5, support the observations seen in the CLSM micrographs, where cancer cells treated with the RRM-MV analogue showed cellular apoptosis and necrosis and changes in cell morphology (Figures 2&3), with no similar cytotoxic effects on normal cell lines (Figure 4).

Interestingly, treatment of the mouse macrophage cell line J774 with RRM-MV or RRM-C appeared to reduce the cytotoxicity levels in these cells, yet, we cannot explain the factors leading to this reduction. The mouse primary fibroblasts were not available at the time when the LDH assay was performed on other cell lines.

### Evaluation of cellular viability in peptide treated cell cultures

#### Effect of a single RRM peptide dose and incubation time on cancer and normal cell lines

**Figure 6:**
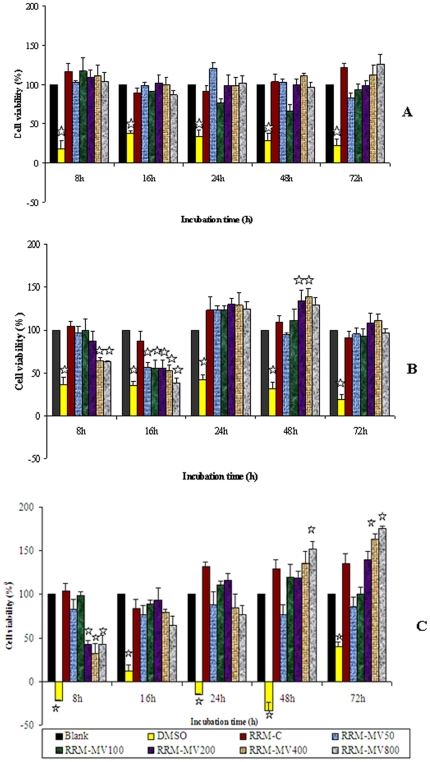
Cellular viability of mammalian cell lines treated with a single dose of different concentrations of the RRM peptides up to 72 h after treatment. **A.** CHO, **B.** B16F0 and **C.** COLO16 cells. Cell cultures (1×10^5^) were incubated for 4 h, 8 h, 16 h, 24 h, 48 h and 72 h with (50 ng/ml, 100 ng/ml, 200 ng/ml, 400 ng/ml and 800 ng/ml) of RRM-MV and 400 ng/ml of RRM-C. A blank (no treatment control) and a positive control (treated with 60% DMSO) were included in all assays. OD was measured at 570 nm after addition of the Prestoblue™ reagent and incubation for 30 min. Cell viability was calculated and is shown relative to that of untreated (blank) sample (set to 100%). Each bar represents mean ± standard errors of 3 separate experiments in triplicate. Data values that are significantly altered (ANOVA and Dunnett's post hoc analysis) when compared to the untreated cells are indicated by the star symbol (*p*<0.05).

The Prestoblue™ reagent was used in an assay to detect cellular viability of a normal cell line (CHO) and two cancer cell lines (B16F0 and COLO16) following treatment with RRM peptides. The cell lines were treated with different concentrations of RRM-MV for up to 72 hours in a single dose. Cellular viability was calculated for cell lines treated with RRM-MV concentrations of 50 ng/ml to 800 ng/ml from 4 h to 72 h. The cell lines responded differently to RRM-MV treatment for both the peptide dose and incubation time as indicated in Figure 6 (A, B &C).

Treatment of normal cells (CHO) with RRM-MV and RRM-C did not have any significant effect on the viability of the cells over the 3 days incubation period with all peptide concentrations (Figure 6 A). On the other hand cellular viability was significantly affected by RRM-MV treatment in both cancer cell lines. The mouse melanoma cells (B16F0) were more resistant to the RRM-MV treatment than the human squamous cell carcinoma cells (COLO16) with a maximum cytotoxic effect for the highest concentrations at 16 hours of incubation for B16F0 while a significant reduction in cell viability was noticed for COLO16 at 8 h of incubation with the 3 highest concentrations used as compared with the effect of RRM-C and both the positive and negative controls (Figure 6, B & C). However, the cell viability for both cell lines increased significantly after 16 h of incubation with the single dose of the peptide indicating the limited effect of a single peptide dose on these cells and the need for a repeated treatment with a second dose.

#### Effect of a repeated dose of the RRM peptides on cellular viability of cancer cells over time

**Figure 7:**
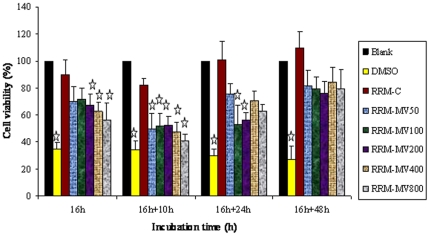
Cellular viability of mouse melanoma cells B16F0 overtime following a repeated dose of the RRM peptides. Cell cultures (1×10^5^) were first treated with 50 ng/ml, 100 ng/ml, 200 ng/ml, 400 ng/ml and 800 ng/ml of RRM-MV and 400 ng/ml of RRM-C for 16 h. A second dose of RRM-C (400 ng/ml) or RRM-MV (50 ng/ml, 100 ng/ml, 200 ng/ml, 400 ng/ml and 800 ng/ml) or DMSO (positive control for cell cytotoxicity) was added after the initial 16 h treatment and cell cultures were then incubated further for another 10 h, 24 h and 48 h. OD was measured at 570 nm after addition and incubation with the Prestoblue™ reagent and cell viability was calculated. Cell viability of all treated samples is shown relative to that of untreated (blank) sample (set to 100%). Each bar represents mean ± standard errors of 3 separate experiments in triplicate. Data values that are significantly altered (ANOVA and Dunnett's post hoc analysis) when compared to the untreated cells are indicated by the star symbol (*p*<0.05).

The B16F0 cell line was subjected to a second dose of similar concentrations of RRM-MV after 16 hours of treatment with a first dose of (50 ng/ml, 100 ng/ml, 200 ng/ml, 400 ng/ml or 800 ng/ml). Cellular viability was measured by Prestoblue reagent at 10 h, 24 h and 48 h after giving the second dose of the peptide (Figure 7). The experimental data with statistical analysis indicated that the second dose of each of (50 ng/ml, 100 ng/ml, 200 ng/ml, 400 ng/ml or 800 ng/ml) RRM-MV given 16 h after the first dose has significantly reduced the B16F0 cellular viability in 26 h (16+10 h). Yet only RRM-MV concentrations of 100 ng/ml and 200 ng/ml introduced a significant reduction in cellular viability at 40 h (16+24 h), and then afterwards reversed to an increase in cellular viability at (16+48 h) for all of the above concentrations of RRM-MV (Figure 7). A comparison of data for cellular viability of B16F0 after incubation for 24 h with a single dose of RRM-MV (Figure 6 B) with the data for cellular viability of the same cell line subjected to a second dose of similar concentrations of RRM-MV at (16+10 h) (Figure 7), indicate that the second dose of the bioactive peptide RRM-MV at 16 h has significantly improved/increased its cytotoxic effect on the B16F0 cells.

Further more, cellular viability was not significantly affected by treatment with 400 ng/ml of the control peptide RRM-C at any time point during this experiment, as indicated by the statistical analysis for our data. However, it is worth to note that cellular viability for cell cultures treated with 400 ng/ml RRM-C has insignificantly increased at (16+48 h), when compared with the non treated cell culture and with RRM-MV treated cultures (Figure 7).

### The effect of RRM-MV on Akt activity by immunoblotting

**Figure 8:**
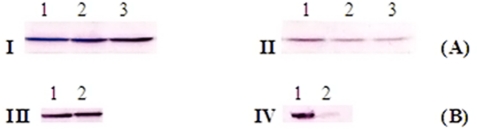
The effect of RRM peptide treatment on total Akt and p- Akt in B16F0 cell line. **A.** Western blots for total Akt (pan) Rabbit MAB in **I** and p-Akt (Thr308) Rabbit MAB in **II** without Akt inhibitor. **B.** Western blots for B16F0 cells treated with 50 µM LY294002 (PI3 kinase inhibitor) prior to immunoblotting with total Akt rabbit MAB in **III** and p-Akt (Thr308) rabbit MAB in **IV**. In **A**, Cells were grown in DMEM only in lane 1; DMEM and 800 ng/ml RRM-C in lane 2; and DMEM with 800 ng/ml RRM-MV in lane 3. In **B**, cells were either grown in DMEM in lane 1, or in DMEM with 50 µM LY294002 for 1 h in lane 2. Similar intensities of the 60 kDa immune bands for total Akt and p-Akt in treated and non treated cells in A indicate the lack of effect of the RRM-designed peptides on p-Akt activity as compared with the inhibitory effect of the Akt inhibitor on p-Akt activity in B.

Western blot results for detection of total Akt in B16F0 cell lysates with rabbit pan-Akt monoclonal antibody indicated the presence of a similar intensity protein band for total Akt (around 60 kDa) in all samples (non treated; treated with 800 ng/ml RRM-C; with 800 ng/ml RRM-MV). This result indicates that the peptide treatment has no significant effect on total Akt protein or on p-Akt (Thr308) (Figure 8 A; I & II). The total Akt activity was not inhibited when the B16F0 cells were treated with 50 µM of the PI3 kinase inhibitor (LY294002). Conversely, the p-Akt immune band was barely detected when the B16F0 cells were treated with 50 µM of LY294002, as compared with B16F0 cells without LY294002 treatment, indicating the specific inhibitory effect of LY294002 on p-Akt in B16F0 cell line (Figure 8 B, III & IV respectively). These results indicate that the Akt signalling pathway in the B16F0 cells does not seem to be affected by the bioactive peptide RRM-MV. In contrast the NM-T5 myxoma virus protein has been found to activate Akt phosphorylation in permissive human cancer cells, by forming a complex between M-T5 and Akt [9].

## Discussion

The RRM approach has been previously utilised in computational analysis of oncogene and proto-oncogene proteins and the results showed that the RRM is capable of identifying the differences between oncogenic and proto-oncogenic proteins with the possibility of identifying the “cancer-causing” features within their protein primary structure [17], [22], [29], [30], [31]. We have recently applied the RRM approach in structure-function analysis of mammalian IL12 proteins and the design of a short therapeutic peptide having murine IL12-like activity [32]. This IL12 peptide analogue (2.18 kDa, 18 aa) induced a dose and time dependent cytotoxic effect on the B16F0 mouse melanoma cell line when assessed by CLSM microscopy and cellular cytotoxicity assays [32].

In the current study we applied the RRM approach to designing RRM-MV, a 2.3 kDa, 18 aa linear peptide with apoptotic bioactive frequencies, to mimic the effect of the 49 kDa M-T5 protein molecule of myxoma virus. RRM-C, a negative control peptide, was similarly designed using the RRM, but lacked the bioactive frequencies of RRM-MV.

The efficacy of RRM-MV peptide as a candidate for cancer therapy was experimentally validated *in vitro* on tumor and on normal cell lines/primary cultures. The cellular cytotoxicity of this bioactive peptide on cancer and normal cell lines was qualitatively confirmed by the fluorescent apoptosis/necrosis assay with CLSM, in addition to the quantitative evaluation of cellular cytotoxicity by LDH assay, and cellular viability by the Prestoblue reagent. Cellular viability of peptide treated cancer cells was compared to cellular viability of peptide treated normal cells. The cytotoxic effects of the bioactive peptide RRM-MV by LDH assay were obvious and significant on the mouse melanoma cells (B16F0) and on the human squamous cell carcinoma (COLO 16), when compared with the effect of the negative control RRM-C on these cell lines and with the non-treated cultures incubated under similar conditions.

When the dose/time -dependent cytotoxic effect of RRM-MV was assessed by the cell viability assay at different peptide concentrations, ranging from 50 ng/ml to 800 ng/ml, for incubation time periods, ranging from 8 h to 72 h, the maximum significant cytotoxic effect of RRM-MV on B61F0 cells was achieved at 16 h after treatment with all peptide concentrations while COLO16 cell line was more affected at 8 h after treatment with similar concentrations indicating that this cell line is more susceptible to RRM-MV treatment. However, the cytotoxicity of the peptide treatment on both cell lines decreased gradually after 24 h which is an expected outcome as the estimated half life of RRM-MV in mammalian reticulocytes in vitro was calculated as 30 h (http://au.expasy.org/tools/protparam.html). Hence a second dose of the RRM-MV given at 16 h after the first dose did significantly enhanced/extended the cytotoxic effect of the peptide treatment over 2 days, indicating the need for a third dose after 48 h. It has also been noticed that the cancer cell lines grew more aggressively after 48 h treatment with the higher concentrations of RRM-MV. Yet, the negative control peptide RRM-C was not cytotoxic to any of the cell lines used in the study at the longest treatment period. Normal cell lines were not significantly affected by the RRM-MV treatment which resulted in negligible damage to normal skin cells, to macrophages and to CHO cells, revealing that this bioactive peptide analogue (RRM-MV) has a selective cytotoxic effect on cancer cells only. When the mouse macrophages were exposed to RRM-MV, there was some indication of apoptosis, but we believe that this is because of the ability of macrophages (as one of the components of the immune system) to uptake the RRM-MV foreign molecule leading to intracellular damage as observed by CLSM. This may also indicate the possible internal effect of RRM-MV on cellular membranes after the effective uptake of the foreign bioactive peptide analogue by the macrophages [33], [34], [35], [36], [37]. The effect of RRM-MV on cellular membranes should be further investigated, as the peptide could be acting on specific internal targets on the cell membrane.

The experimental data on the Akt cell signalling pathway [38], [39] in RRM-MV treated cancer cells, indicated that p-Akt levels expressed by B16F0, were not affected by the treatment with RRM-MV as compared with the non-treated controls. It is known that the direct interaction between NM-T5 and Akt is the key for MV tropism in some human cancer cell lines, and that the level of phosphorylated Akt can be affected by this viral protein [9], [10]. It does not seem that RRM-MV is targeting the serine/threonine kinase (Akt) pathway [33], [38] to induce cellular cytotoxicity. Therefore, other possible apoptotic cell signalling targets for RRM-MV in human cancer cell lines are currently under investigation by our team in addition to specific markers on cellular membranes of cancer and normal cells.

By applying the RRM model we were able to design a single short peptide with high levels of specificity and apoptotic activity against cancer cells, yet the same peptide has a negligible toxic effect on normal cells. This is a unique illustration of utilising the RRM to design bioactive peptides with specific tumor cell cytotoxicity, indicating that RRM has the potential to be applied in designing new, novel peptide therapeutics.
